# The Interplay of Notch Signaling and STAT3 in TLR-Activated Human Primary Monocytes

**DOI:** 10.3389/fcimb.2018.00241

**Published:** 2018-07-10

**Authors:** Dagmar Hildebrand, Florian Uhle, Delal Sahin, Ute Krauser, Markus Alexander Weigand, Klaus Heeg

**Affiliations:** ^1^Centre for Infectious Diseases, Medical Microbiology and Hygiene, Heidelberg University Hospital, Heidelberg, Germany; ^2^Department of Anesthesiology, Heidelberg University Hospital, Heidelberg, Germany

**Keywords:** infection, Notch signaling, DLL1, STAT3, TLR, PD-L1

## Abstract

The highly conserved Notch signaling pathway essentially participates in immunity through regulation of developmental processes and immune cell activity. In the adaptive immune system, the impact of the Notch cascade in T and B differentiation is well studied. In contrast, the function, and regulation of Notch signaling in the myeloid lineage during infection is poorly understood. Here we show that TLR signaling, triggered through LPS stimulation or *in vitro* infection with various Gram-negative and -positive bacteria, stimulates Notch receptor ligand Delta-like 1 (DLL1) expression and Notch signaling in human blood-derived monocytes. TLR activation induces DLL1 indirectly, through stimulated cytokine expression and autocrine cytokine receptor-mediated signal transducer and activator of transcription 3 (STAT3). Furthermore, we reveal a positive feedback loop between Notch signaling and Janus kinase (JAK)/STAT3 pathway during *in vitro* infection that involves Notch-boosted IL-6. Inhibition of Notch signaling by γ-secretase inhibitor DAPT impairs TLR4-stimulated accumulation of NF-κB subunits p65 in the nucleus and subsequently reduces LPS- and infection-mediated IL-6 production. The reduced IL-6 release correlates with a diminished STAT3 phosphorylation and reduced expression of STAT3-dependent target gene programmed death-ligand 1 (PD-L1). Corroborating recombinant soluble DLL1 and Notch activator oxaliplatin stimulate STAT3 phosphorylation and expression of immune-suppressive PD-L1. Therefore we propose a bidirectional interaction between Notch signaling and STAT3 that stabilizes activation of the transcription factor and supports STAT3-dependent remodeling of myeloid cells toward an immuno-suppressive phenotype. In summary, the study provides new insights into the complex network of Notch regulation in myeloid cells during *in vitro* infection. Moreover, it points to a participation of Notch in stabilizing TLR-mediated STAT3 activation and STAT3-mediated modulation of myeloid functional phenotype through induction of immune-suppressive PD-L1.

## Introduction

The highly conserved Notch signaling pathway has virtually a simple composition and depends on Notch ligands binding to Notch receptors on neighboring cells. In mammals, there are four Notch receptors (Notch1-4) and five Notch ligands [Jagged-1, Jagged-2, Delta-like 1 (DLL1), DLL3, and DLL4] (Radtke et al., 2010). Both, Notch receptor and ligands are transmembrane proteins with large extracellular domains. Upon ligand binding, a conformational change in the receptor enables two consecutive proteolytic processing events. The first cleavage, that results in shedding of the extracellular domain, is mediated by the ADAM (disintegrin and metalloproteases)-family. The following cleavage inside the transmembrane domain is catalyzed by a multicomponent γ-secretase complex that releases Notch intracellular domain (NICD) to translocate to the nucleus. In the nucleus, the NICD interacts with the DNA-binding protein CSL (also termed RBP-J) that unbound probably functions as transcriptional repressor. Binding of NICD displaces co-repressor complexes, recruits co-activators (mastermind proteins), and finally induces transcription of Notch target genes (Kopan and Ilagan, 2009; Kovall and Blacklow, 2010; Bray, 2016). In mammals, the most common Notch target genes are members of the basic-helix-loop-helix transcription factors belonging to the hairy and enhancer of split (HES) and HES with YRPW motif (HEY) families (Iso et al., 2003). Similar to their receptors, Notch ligands also undergo ADAM-and γ-secretase-mediated cleavage upon receptor-binding (Zolkiewska, 2008).

The induction of Notch target genes contributes to a wide array of developmental processes in different organ systems. Notch engages a central role in the hematopoietic as well as the immune system through regulating multiple lineage decisions of developing immune cells (Radtke et al., 2010). Particularly in the adaptive immune system, the impact of the Notch cascade in T and B differentiation is well accepted (Radtke et al., 2004). For a long time, myeloid cells such as monocytes, macrophages and dendritic cells (DCs) were mostly considered as signal-sending cells that express Notch ligands and activate the cascade in receptor expressing, signal-receiving lymphocytes. Nevertheless, also myeloid cells do express Notch receptors and the influence of Notch on myeloid cell differentiation becomes more and more apparent (Radtke et al., 2010).

Beside its role in differentiation, increasing evidence suggests an association of Notch signaling in mature immune cell activation and function during viral and bacterial infection (Shang et al., 2016). During infection myeloid cells recognize pathogen-associated molecular patterns (PAMPs) via a variety of pattern recognition receptors (PRRs) including Toll-like receptors (TLRs). In terms of Gram-negative bacteria, the cell wall component lipopolysaccharide (LPS) activates TLR4 that mediates expression of inflammatory cytokines through MAPKinase and Nuclear factor κB (NF-κB) signaling. Subsequently, the released cytokines bind the corresponding receptors in an autocrine and paracrine fashion, activate signaling cascades such as the JAK/STAT pathway and thereby modulate the further direction of the immune response.

As an inflammatory environment is associated with Notch signaling a modulation of the cascade through TLR-mediated signaling seems likely but is far from being elucidated. Activated TLR4-signaling might modulate the Notch cascade directly through histones modifications at the Notch target gene loci (Hu et al., 2008) and indirectly through induction of Notch receptors and ligands (Monsalve et al., 2006; Palaga et al., 2008). On the other hand, activated Notch signaling might modulate TLR signaling. However, whether the modulation is supportive or inhibitory is controversially discussed (Monsalve et al., 2006, 2009; Zhang et al., 2012).

In this study, we set out to investigate the regulation of Notch signaling in TLR-activated primary monocytes and clarify the interaction between both cascades. Our data show that LPS-stimulation and *in vitro* infection with Gram-negative and Gram-positive bacteria stimulates expression of Notch receptor ligand DLL1 and induction of Notch target genes in primary human monocytes. Furthermore, DLL1 is strongly upregulated in LPS-stimulated systemic inflammation in mice. The TLR4-stimulated production of DLL1 seems to be an indirect effect, provoked by TLR-stimulated cytokines and the subsequent activation of the transcription factor STAT3. Our data support the hypothesis that Notch signaling increases TLR4-stimulated inflammatory responses of myeloid cells as inhibiting NICD through γ-secretase inhibitor (GSI) DAPT impaired IL-6 expression of activated monocytes. Thereby infection stimulated activation would provoke a TLR-signaling primed positive feedback loop between the Notch cascade and STAT3 that sustains the activity of the key transcription factor. Finally, we propose that Notch-mediated stabilization of STAT3 activity participates in remodeling the functional phenotype of monocytes through facilitating STAT3-dependent target genes, such as immuno-suppressive PD-L1.

## Results

### TLR-activation stimulates notch ligand DLL1 expression in human primary monocytes and mice

To investigate the regulation of Notch signaling in TLR-activated myeloid cells, primary monocytes isolated from blood of healthy donors were infected with Gram-positive *Enterococcus faecalis* or different Gram-negative bacteria (GN), namely *Escherichia coli, Klebsiella pneumonia*, and *Pseudomonas aeruginosa*. Furthermore, cells were stimulated with LPS, the main component of GN outer membrane and virulence factor that activates TLR4 signaling (Chow et al., 1999). Bacteria were killed by gentamicin 2 h after infection. The next day supernatant and infected/LPS-treated cells were analyzed.

**Figure 1:**
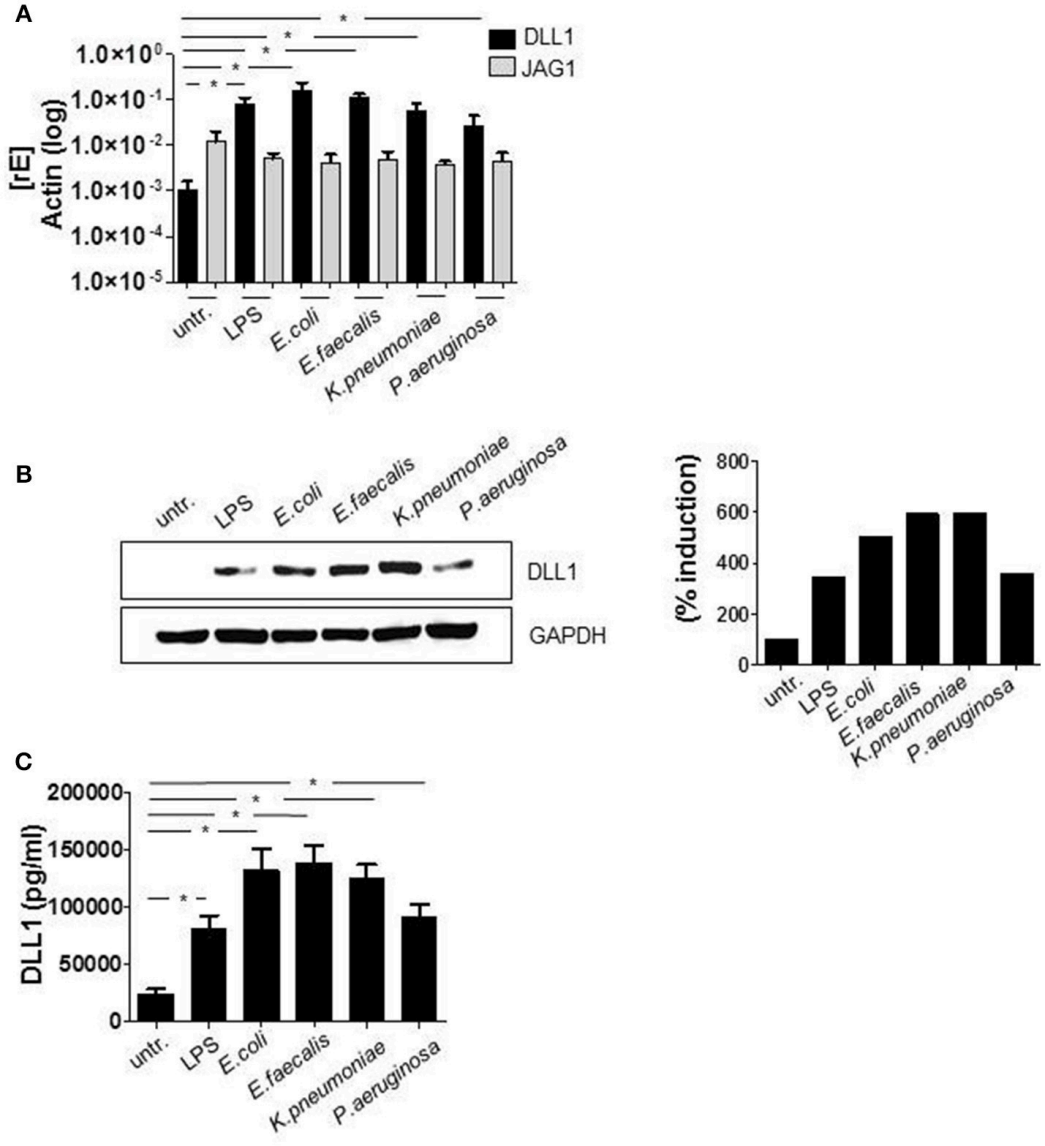
TLR signaling induces DLL1 in primary human monocytes. CD14^+^ monocytes, isolated from blood of healthy donors were stimulated with 100 ng/ml LPS or infected with *Escherichia (E.) coli, Enterococcus (E.) faecalis, Klesiella (K.) pneumoniae, Pseudomonas (P.) aeruginosa* in a concentration of 10^6^ bacteria per 10^6^ monocytes/ml. After 2 h bacteria were killed by gentamicin. The next day cells and supernatant were analyzed. **(A)** RNA was isolated and cDNA produced. Induction of gene expression was analyzed by qRT PCR using sequence-specific primer for DLL1 and JAG1 (gene encoding Jagged-1) and SYBR Green Master mix. Actin was detected as endogenous control for normalization. **(B)** For western blot analysis equal amounts of protein lysates were blotted and probed with antibodies against DLL1 or GAPDH (loading control). Shown is one representative blot and the associated quantification. Quantification was performed using the Image Analysis System Bioprofil (Fröbel, Germany). The intensity of signals were calculated against loading control and presented as percent of untreated samples. **(C)** Supernatants were used for ELISA analysis to quantify shedded DLL1 extracellular domain. **(A,C)** depict the mean and standard deviation of at least three donors. Statistics: ^*^p ≤ 0.05 by Mann–Whitney *U*-test.

**Figure 2:**
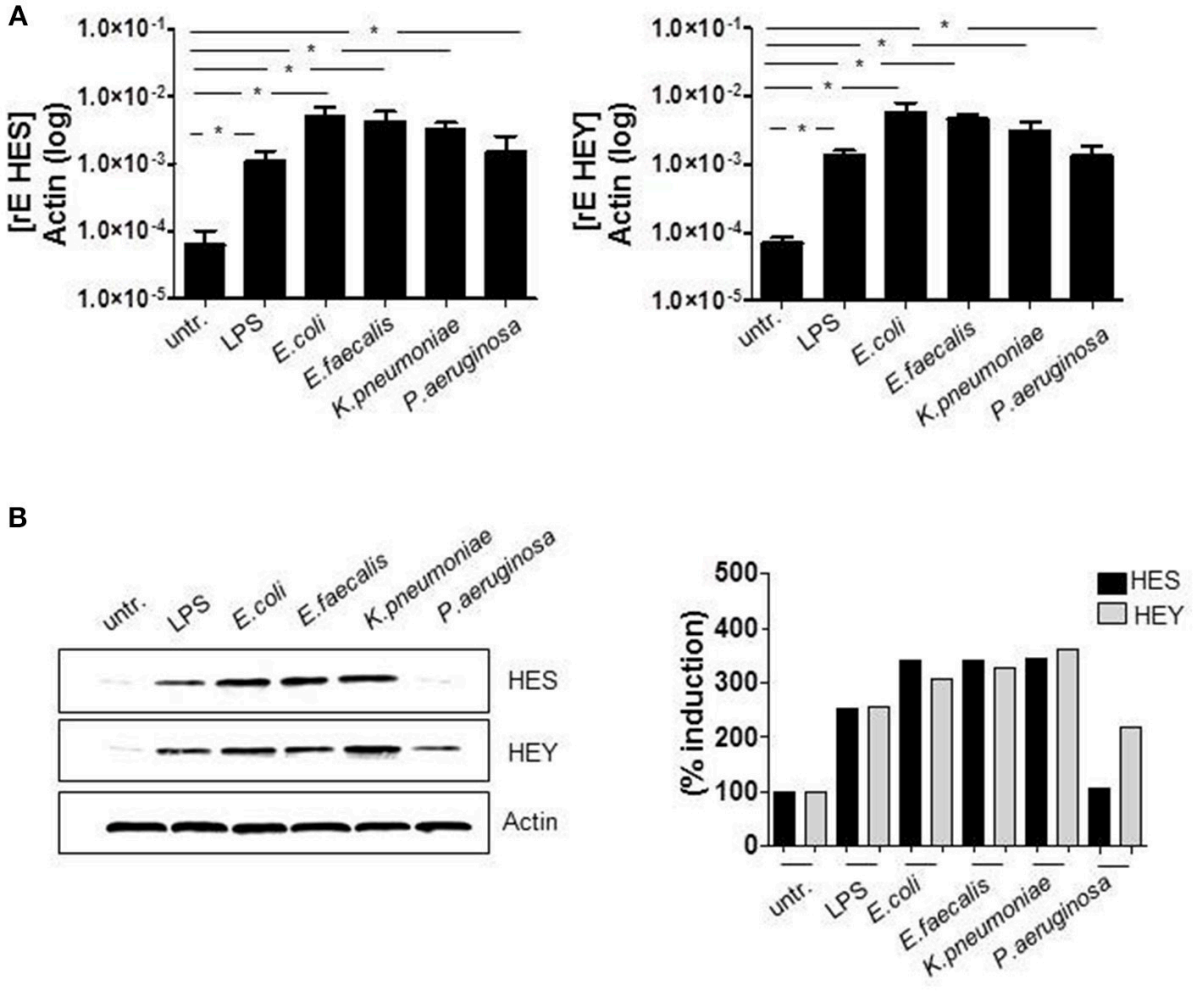
TLR activation induces Notch signaling. Primary human monocytes were treated as described in Figure 1. **(A)** HES and HEY gene expression was analyzed by qRT PCRs. **(B)** Protein expression was detected by western blot analyses. Results were quantified. **(A)** Shows the mean and standard deviation of three donors. Statistics: ^*^p ≤ = 0.05 by Mann–Whitney *U*-test.

Initially, we checked the induction of Delta-like (DLL) and Jagged Notch ligands after TLR activation. Although the regulation of the Notch cascade is complex, induction of Notch ligands is one essential part of the control network and therefore important to determine. The qRT PCR data in Figure 1A show that Delta-like and Jagged ligands seem to be differentially regulated. In comparison to DLL1, the basal expression of JAG1 (gene encoding Jagged-1) is elevated in untreated monocytes. Twenty-four hours after TLR activation through *in vitro* infection or LPS stimulation, the expression of JAG1 decreases, whereas DLL1 gene expression is highly induced (Figure 1A) and translated into the protein DLL1 (Figure 1B). Additionally performed ELISA analyses confirmed shedding of the DLL1 extracellular domain into the culture supernatant (Figure 1C). The release of cleaved DLL1 occurs upon Notch receptor binding. Therefore one can conclude binding of the ligand to the respective DLL1 receptor. Figure 2A confirms this presumption and reveals a significant induction of Notch signaling target genes HES and HEY after TLR activation. Western blot analyses (Figure 2B) and the associated quantifications further show that HES and HEY mRNAs are translated into the respective proteins.

**Figure 3:**
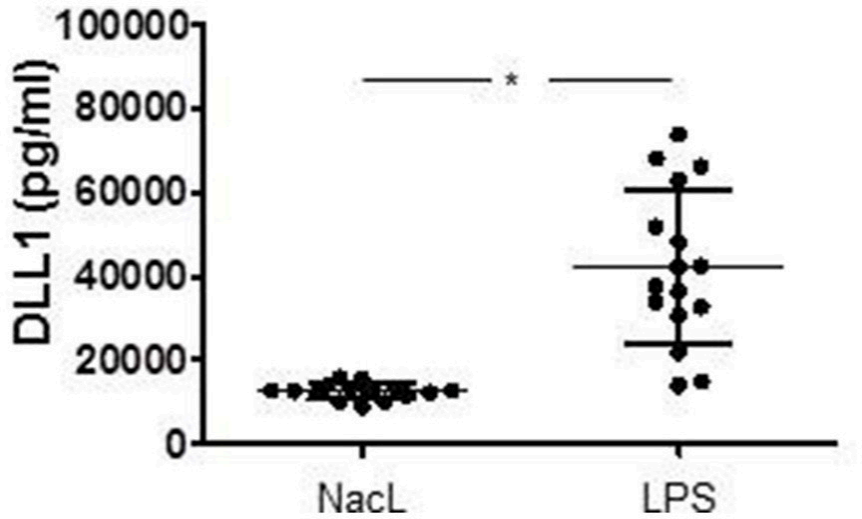
LPS-induced systemic inflammation in mice. Twelve week old male mice were injected intraperitoneally with LPS or NaCL (control group). After 24 h blood were taken and analyzed for DLL1 by ELISA analyses. Statistics: ^*^p ≤ = 0.05 by Mann–Whitney *U*-test.

In order to verify, whether TLR signaling stimulates DLL1 expression and shedding in an *in vivo* situation, we induced systemic inflammation in mice by injecting LPS (endotoxin mouse model). In our model, 12 week old male C57BL/6 mice were injected intraperitoneally with LPS or NaCl (control group) (*n* = 16 each group). After 24 h, blood was taken and analyzed for DLL1 by ELISA. Figure 3 compares the DLL1 plasma level of LPS-injected and control mice and reveals a pronounced and significant higher concentration of the Notch ligand in mice with systemic inflammation.

### TLR-signaling promotes DLL1 expression indirectly through cytokine receptor- triggered STAT3 activation

**Figure 4:**
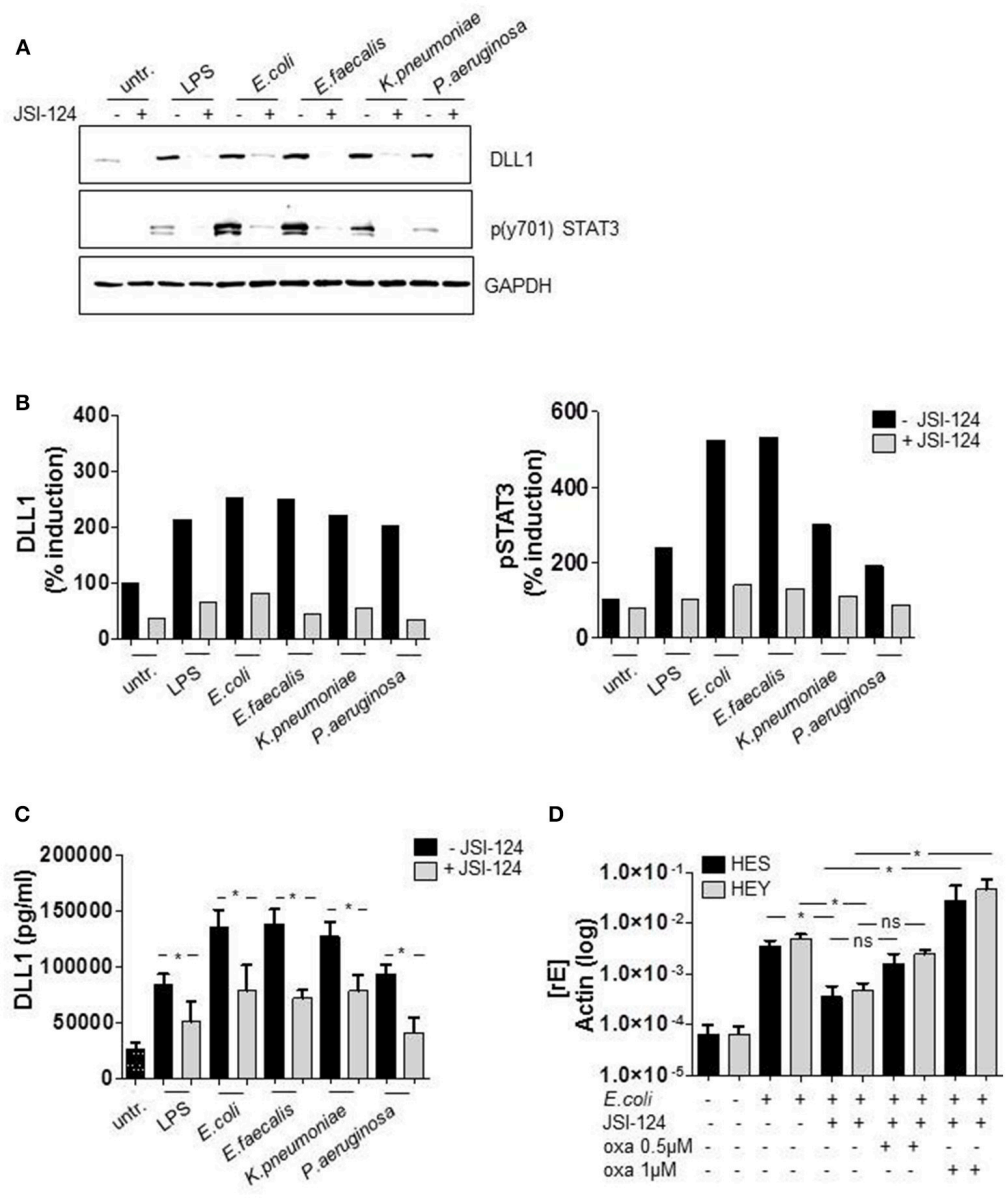
TLR4-signaling promotes DLL1 expression through STAT3. Human blood-derived monocytes were pretreated with STAT3 Inhibitor JSI-124 (200 nM) for 2 h before stimulation with LPS or infection. **(A)** The next day lysates were produced and applied for western blot analyses for the detection of phosphorylated (Tyr 701) STAT3 and DLL1. GAPDH was detected as loading control. The experiment was repeated two times with comparable results. **(B)** Quantification of **(A)**. **(C)** Supernatant was analyzed by ELISA for shedded DLL1. **(D)** JSI-124 pretreated and *E. coli*-infected cells were stimulated with 0.5 or 1 μM oxaliplatin (oxa). The next day cDNA was produced and analyzed for HES and HEY induction. **(C,D)** mean ± std *n* = 3, ^*^p ≤ = 0.05 by Mann–Whitney *U*-test.

As we had observed a pronounced TLR ligand-stimulated expression of DLL1 and Notch target genes, we aimed to discover the underlying mechanism. Whereas several publications show that TLR ligands can induce Notch components, the actual signaling behind that induction has not been clarified yet. For TLR4-induced DLL1 expression an indirect, not further specified mechanism was suggested (Foldi et al., 2010). Therefore we screened for predicted transcription factor binding sites in the DLL1 promotor region and identified STAT3 as a promising candidate (www.genecards.org/cgi-bin/carddisp.pl?gene$=$DLL1). The putative STAT3 binding sites in the promoter region of DLL1 are depicted in Supplementary Figure 1. The western blot in Figure 4A and the associated quantification (Figure 4B) show that STAT3 is highly phosphorylated in LPS-stimulated as well as *in vitro* infected monocytes. Pretreatment of cells with the STAT3 specific inhibitor JSI-124 for 2 h effectively suppressed the activation of STAT3 and the diminished activation correlates with a reduction of DLL1 expression in the activated monocytes (Figures 4A,B). Also, levels of shedded DLL1 in the supernatant were significantly reduced by inhibiting STAT3 in cells stimulated by different bacteria (Figure 4C). The expression of JAG1 was not affected by the inhibitor (Supplementary Figure 2). Till this point, we did not observe any significant differences within the group of Gram-negative bacteria or between Gram-negative and -positive bacteria. Therefore, we concentrated on *E. coli*-infection and TLR4-mediated signaling for the subsequent experiments. In line with the previous results, *E. coli*-stimulated Notch target genes HES and HEY were also affected by STAT3 inhibitor treatment and additional stimulation with oxaliplatin that activates Notch signaling via activation of the γ-secretase rescued HES and HEY induction (Figure 4D). This supports the hypothesis of an indirect TLR-stimulated activation of the Notch target genes and points to STAT3-dependent DLL1 gene induction.

### Notch signaling augments TLR4-stimulated IL-6 expression

**Figure 5:**
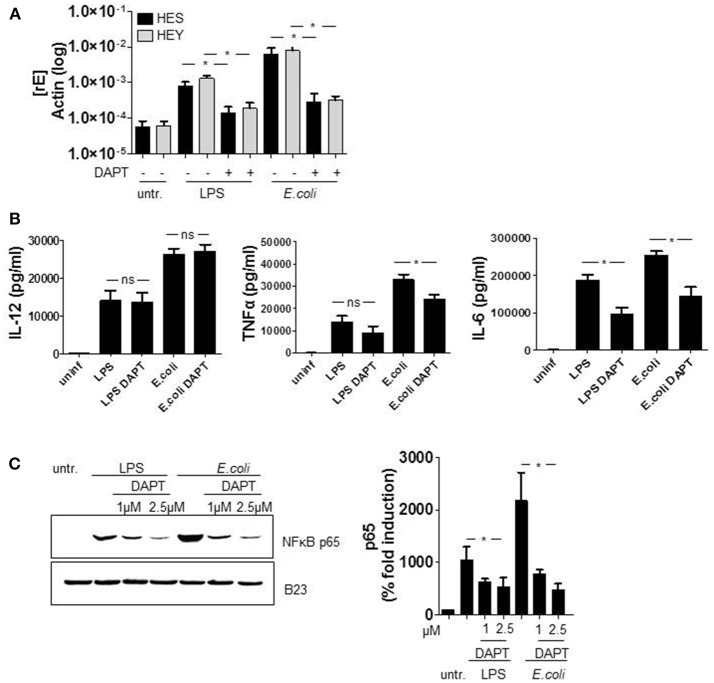
Notch signaling augments TLR4-stimulated IL-6 and TNFα expression. Monocytes were pretreated with DAPT (2.5 μM) for 1 h before LPS stimulation and *E. coli* infection. 2 h after infection, medium was changed and DAPT was added again. **(A)** The next day RNA was isolated, cDNA produced and gene expression was analyzed by qRT PCR using sequence-specific primer for HES and Hey and SYBR Green. Results were normalized against actin. **(B)** Supernatants were used for ELISA analysis to quantify released IL-6, IL-12p40, and TNFα. **(C)** For western blot analysis cells were harvested 2 h after infection. Nuclear lysates were produced and equal amounts of lysates were blotted and probed with antibodies against p65 or B23 (control). Shown is one representative experiment out of three and the associated quantification of three experiments. **(A–C)** mean ± std *n* = 3, ^*^p ≤ = 0.05 by Mann–Whitney *U*-test.

Several publications propose a bidirectional crosstalk of TLR and Notch signaling pathways in macrophages. However, whether the Notch cascade impairs or augments TLR-mediated activation and production of pro-inflammatory cytokines is controversially discussed (Palaga et al., 2008; Monsalve et al., 2009; Zhang et al., 2012). To check an influence of Notch in LPS- and *E. coli*-stimulated cytokine production in primary human monocytes, cells were treated with the γ-secretase inhibitor (GSI) DAPT. Figure 5A confirms the abrogation of Notch signaling through DAPT as HES and HEY gene expression was suppressed by the inhibitor. The further performed ELISA analysis revealed that GSI treatment had no effect on TLR4*-*stimulated production of IL-12p40, slightly reduced TNFα expression but considerably inhibited IL-6 production to nearby sixty percent (Figure 5B).

TLR4-activation stimulates cytokine expression amongst other cascades through NF-κB signaling. As a Notch-mediated modulation of NF-κB was reported previously in T cells (Shin et al., 2006; Vacca et al., 2006; Vilimas et al., 2007), we set out to investigate a potential involvement of NF-κB in the DAPT-mediated reduction of cytokine expression. Therefore we treated the cells once more with DAPT and stimulated TLR4 signaling for 2 h with either LPS or *E. coli*. The subsequently produced nuclear fraction of lysed cells we analyzed for the amount of NF-κB subunit p65 which is known to induce IL-6 gene expression. The western blot in Figure 5C and the associated quantification of results clearly reveal that inhibiting Notch signaling reduces the level of p65 in the nucleus of LPS- and *E. coli*-stimulated monocytes. This strongly suggests that NICD-modulated NFκB signaling accounts for the decreased IL-6 production in Notch inhibited monocytes.

### Notch signaling activates STAT3 and mediates STAT3 target gene expression

**Figure 6:**
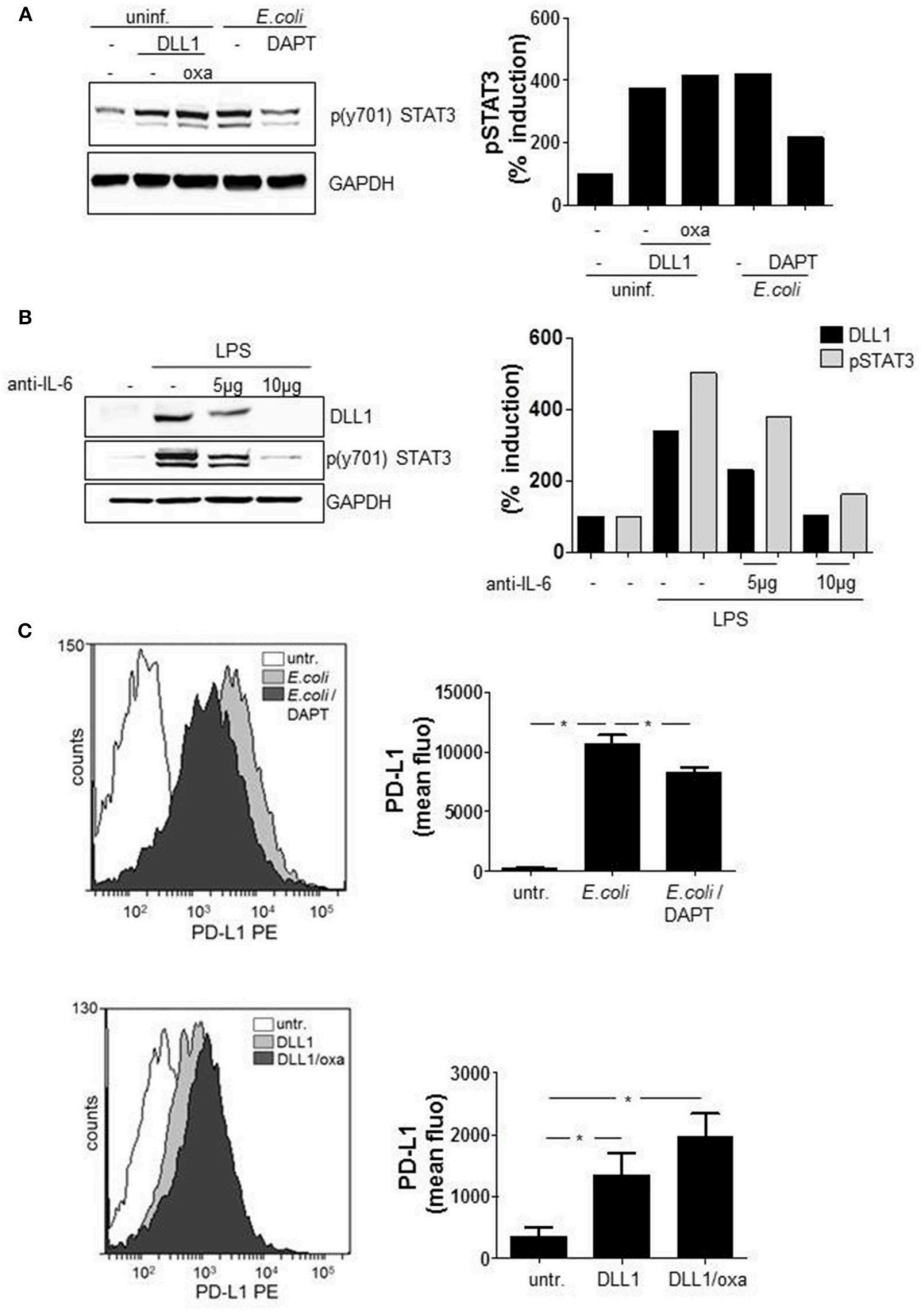
Notch signaling modulates functional myeloid phenotype. Primary blood-derived monocytes were stimulated with 3 μg/ml recombinant soluble DLL1 ± 1 μM oxaliplatin (oxa). Aside from that cells were treated with DAPT (1 μM) 1 h before infection with *E. coli*. **(A)** Cell lysates were analyzed by western blots for STAT3 phosphorylation. GAPDH was detected as loading control. Results were quantified. **(B)** Monocytes were treated with an anti-IL6 specific antibody (Thermo Fisher Scientific) in parallel to LPS (100 ng/ml) stimulation. The next day lysates were produced and used in western blot analyses for detection of p(y701)STAT3 and DLL1. Loading control: GAPDH. Experiment was repeated two times with comparable results. **(C)** After 3 days cells were analyzed with fluorescently-labeled antibodies by flow cytometry for surface expression of PD-L1. Shown are FACS histogram overlays (Weasel.jar software) and the associated quantification of three experiments. Mean ± std *n* = 3, ^*^p ≤ = 0.05 by Mann–Whitney *U*-test.

According to our results, STAT3 induces DLL1 and DLL1-mediated Notch signaling boosts TLRL-stimulated IL-6. As binding of IL-6 to the respective receptor mediates activation of the JAK2/STAT3 cascade a positive feedback loop between the Notch pathway and STAT3 seemed likely. To test this hypothesis, we activated Notch signaling in primary monocytes and analyzed activation of STAT3. The western blot in Figure 6A confirms that treatment with recombinant soluble DLL1 (extracellular domain) stimulates STAT3 phosphorylation and reveals that the additional boost of Notch signaling through oxaliplatin (“oxa”) further slightly enhanced the activation. As additional hint for a positive feedback loop, inhibition of Notch signaling with DAPT resulted in reduced *E. coli*-stimulated phosphorylation of STAT3 (Figure 6A). Finally, blocking IL-6 receptor signaling through an anti-IL6 blocking antibody reduced TLR4-mediated STAT3 phosporylation and DLL1 expression (Figure 6B).

STAT3 is known to be one main regulator of the functional phenotype of myeloid cells (Cheng et al., 2003; Melillo et al., 2010; Dufait et al., 2016; Giesbrecht et al., 2017). As inducer of several T cell-inhibiting factors such as PD-L1, the transcription factor is considered as a mediator of an immuno-suppressive APC phenotype. Recently we showed that PRR-mediated pro-inflammatory cytokines activate the key transcription factor STAT3 that ultimately induces a shift in gene expression and induction of several T cell suppressive factors (Wolfle et al., 2011; Giesbrecht et al., 2017). Therefore, we addressed the question, whether Notch signaling boosted IL-6 and stabilization of STAT3 activity influences the functional phenotype of monocytes. We evaluated whether activation of Notch signaling results in expression of STAT3-dependent PD-L1. By flow cytometry analysis the surface expression of PD-L1 was quantified on GSI pretreated and *E. coli*-infected monocytes. Additionally, PD-L1 expression was quantified after sDLL1 ± oxaliplatin-stimulation that activates Notch signaling. The flow cytometry data (overlay and associated quantification) of Figure 6C illustrates that *E. coli*-infection stimulates a pronounced upregulation of PD-L1 that is significantly diminished through Notch inhibition by DAPT. Furthermore, treatment with sDLL1 stimulated upregulation of the immuno-checkpoint which further increases after boosting of Notch cascade through oxaliplatin (Figure 6C).

**Figure 7:**
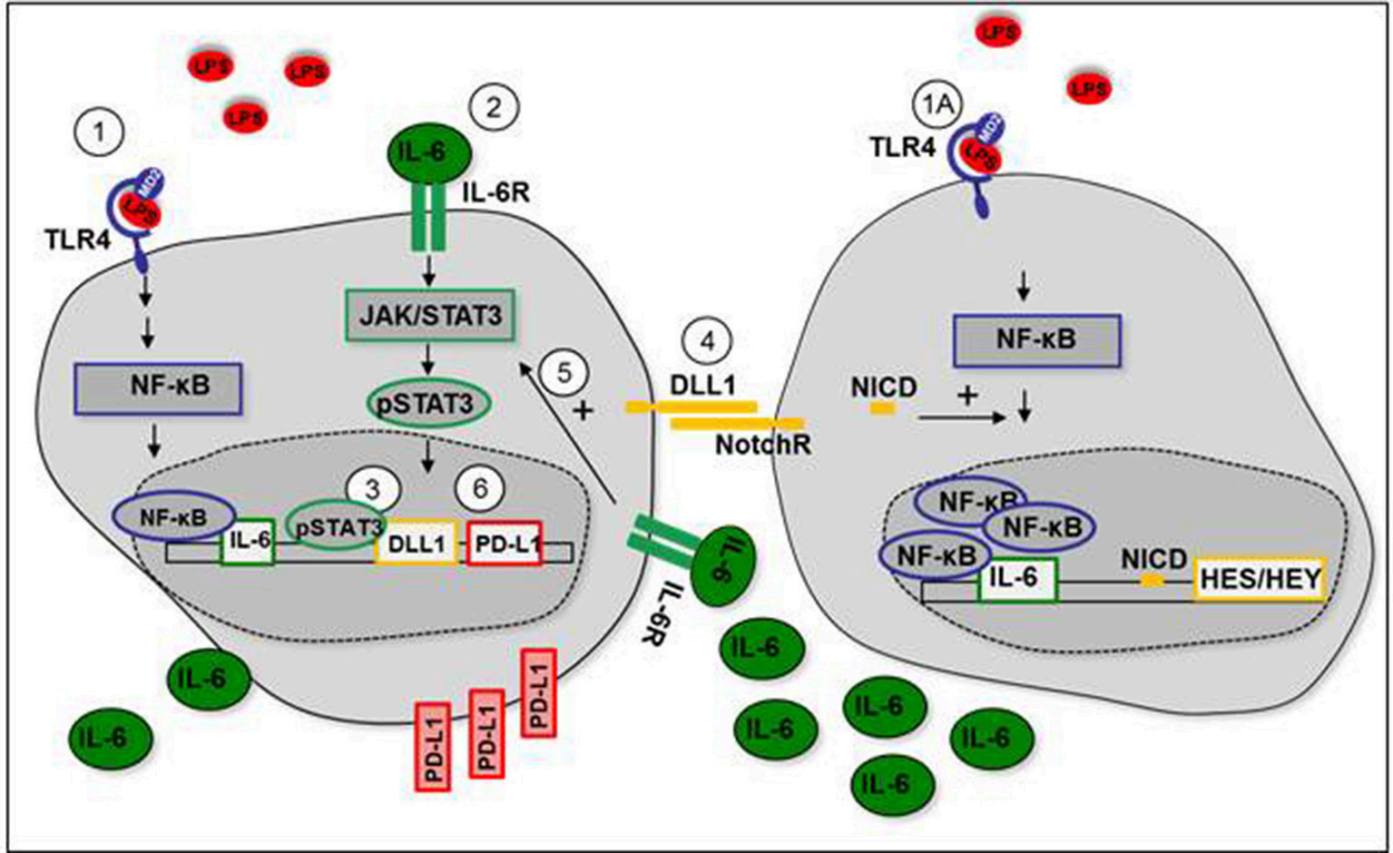
Positive feedback loop between Notch signaling and STAT3 after TLR-activation. (1+1A) LPS stimulates IL-6 production through TLR4-mediated NF-κB signaling. (2) IL-6 binds to IL-6 receptor (IL-6R) and stimulates activation of STAT3. (3) STAT3 induces expression of DLL1. (4) Transmembrane DLL1 binds to Notch receptor on neighboring cells. Notch receptor intracellular domain (NICD) translocates to the nucleus, induces HES/HEY transcription, and mediates NF-κB accumulation in the nucleus. IL-6 production is enhanced. (5) Enhanced IL-6 receptor signaling stabilizes STAT3 activation. (6) STAT3-dependent PD-L1 expression is increased.

Figure 7 summarizes our so far presented results and depicts our proposed mechanism of TLR-stimulated DLL1 and the positive feedback interaction between Notch signaling and STAT3 that results in upregulation of immuno-suppressive PD-L1.

## Discussion

The regulation and function of Notch signaling in myeloid cells involved in innate immunity is poorly understood. Nevertheless, recent findings elucidate a role for the Notch pathway during TLR-associated and inflammation-induced monocyte differentiation and macrophage activation (Radtke et al., 2010; Nakano et al., 2016; Singla et al., 2017). Cells of the myeloid lineage do express a broad range of TLRs as well as Notch receptors and ligands. As both pathways are connected to inflammation, simultaneous activation, and a bidirectional modulation seem likely. However, the reciprocal modulation is far from being understood.

In terms of TLR-mediated regulation of Notch signaling it was shown that the expression of Notch receptors and ligands on myeloid cells of both human and mouse origin can be enhanced in response to TLR ligands (Foldi et al., 2010; Radtke et al., 2010) and infection (Narayana and Balaji, 2008; Ito et al., 2009). Nevertheless, the mechanism of TLR-stimulated induction of Notch ligands remains largely elusive and direct and indirect ways are suggested. A study from Foldi et al. proposes an indirect TLR-mediated induction of Delta-like ligands that seems to be independent of Notch signaling components (Foldi et al., 2010). Our study, in human primary monocytes, confirms the indirect induction of DLL1 and extends the knowledge of the underlying mechanism. According to our experiments with a STAT3-specific inhibitor, the TLR-stimulated expression of DLL1 is dependent on the cytokine receptor-activated transcription factor. As the DLL1 promoter contains a binding site for STAT3 we assume a direct induction. A dependency of DLL1 transcription on STAT family members was reported previously in Influenza A Virus (H1N1) infection. The study in murine bone marrow-derived macrophages shows that H1N1 infection stimulates the autocrine activation of INFαR-signaling, that subsequently mediates STAT1/2-controlled transcription of DLL1 (Ito et al., 2011). For bacterial infection, we propose for the first time a dependency of DLL1 transcription on STAT3 that is activated through TLR-induced cytokines and autocrine cytokine receptor signaling.

In addition to TLR-stimulated Notch pathway modulation, our study highlights the positive regulation of TLR-activated NF-κB signaling and cytokine expression through Notch. In the literature, the Notch-mediated modulation of NF-κB in the myeloid system is discussed controversially. Constitutively active NICD was shown to increase TLR-associated inflammatory response in the murine macrophage cell line Raw 647 and DLL4 was reported to increase LPS-stimulated cytokines by enhancing NF-κB activation (Monsalve et al., 2006, 2009). Contrary to these findings, other studies propose that TLR-mediated pro-inflammatory cytokines are reduced upon over-expression of NICD in mouse peritoneal macrophages (Zhang et al., 2012). Our study supports the hypothesis of a Notch-transduced amplification of inflammation during infection as it reveals a gain of TLR4-primed and NF-κB -mediated expression of IL-6 and TNFα through Notch activation.

Furthermore, we state that Notch-boosted IL-6 and the subsequent autocrine IL-6 receptor signaling, that is known to activate JAK/STAT pathway (Heinrich et al., 2003) stabilize STAT3 activation. In our hands, treatment with recombinant soluble DLL1 stimulates STAT3 phosphorylation in monocytes and the GSI- induced decrease in IL-6 production correlates with a diminished STAT3 phosphorylation. An influence of Notch signaling on STAT3 was observed in the absence of infection in breast cancer cells (Jin et al., 2013) with hyperactivated Notch signaling. According to the data the Notch-induced increase in IL-6 results in autocrine and paracrine activation of JAK/STAT signaling and an upregulation of STAT3-target genes (Jin et al., 2013).

Here we propose a positive feedback loop between STAT3 and DLL1-activated Notch signaling after TLR-mediated inflammation. In our hypothesis, TLR-mediated NF-κB signaling stimulates production of IL-6, which binds to the IL-6 receptor and activates STAT3. STAT3 induces DLL1 expression that activates Notch signaling and boosts NF-κB-induced IL-6 which transduces stabilization of STAT3 activation.

STAT3 is known to be a key transcription factor in immuno-suppression through the direct induction of immunosuppressive factors such as PD-L1 (Wolfle et al., 2011; Giesbrecht et al., 2017). From our previous studies it is known that PRR-mediated inflammatory cytokines remodel myeloid cells toward an immune suppressive phenotype through prolonged activation of STAT3 (Wolfle et al., 2011; Giesbrecht et al., 2017). Here we propose that the positive feedback loop between Notch and STAT3 after TLR activation facilitates activation of the key transcription factor and thereby induction of PD-L1. We show that inhibition of Notch decreases TLR-stimulated PD-L1 surface expression and that recombinant soluble DLL1 increases PD-L1 on monocytes significantly. PD-L1 induces differentiation of regulatory T cells (Tregs) through binding PD-1 on T cells (Francisco et al., 2009). In terms of Treg differentiation, it was shown that DLL1 enhances the conversion of human memory CD4 T cells into Tregs and that the Notch ligand expands FOXP3 positive T cells (Mota et al., 2014). Here we hypothesize that DLL1 and Notch signaling influence adaptive immunity not only directly but also indirectly through STAT3-driven remodeling of myeloid cells toward an immune-suppressive myeloid phenotype that eventually facilitates Treg formation.

In summary our study extends the knowledge of TLR4-mediated regulation of Notch signaling, reveal a bidirectional interaction between Notch signaling and STAT3 and gives first hints to an involvement of Notch in the functional phenotype of monocytes.

## Materials and methods

### Isolation of primary human monocytes

PBMCs were isolated from fresh blood or buffy coat from healthy donors by density gradient centrifugation (Biocoll separating solution, 1.077 g/ml, Biochrom AG, Berlin, Germany). CD14^+^ cells were magnetically labeled with beads (MiltenyiBiotec) and selected via the autoMACS separator (autoMACS, program: possel, Miltenyi Biotec, Bergisch Gladbach, Germany) twice. Purified monocytes (1 × 10^6^ cells/ml) were cultured in RPMI 1640 (Sigma-Aldrich, Taufkirchen, Germany) supplemented with 100 IU/ml of penicillin, 100 μg/ml streptomycin and 10% heat inactivated fetal calf serum (Promocell, Heidelberg, Germany) at 37°C in a humidified atmosphere in the presence of 5% CO_2_.

### Treatment of cells

Monocytes were stimulated with 100 ng/ml LPS, kindly provided by U.Seydel (Borstel). For inhibition of STAT3 activation cells were treated with 200 nM, JSI-124 (Calbiochem, Schwalbach, Germany), 2 h prior infection/LPS stimulation. For Notch signaling activation monocytes were stimulated with 3 μg/ml recombinant soluble DLL1 (PeproTech, Hamburg, Germany) ±1 μM Oxaliplatin (BioCat GmbH, Heidelberg, Germany) for the indicated timepoints. For inhibition of Notch signaling cells were pretreated (1 h) with 2.5 μM DAPT (Sigma-Aldrich, Taufkirchen, Germany).

### *In vitro* infection

*Escherichia coli (*ATCC25922)*, Klebsiella pneumonia (*ATCC700603)*, P. aeruginosa (*PA01) *and E. faecalis (*ATCC29212) were cultured overnight on Columbia blood sheep agar at 37°C at 5% CO_2_ in a humidified atmosphere. The next day 1 colony of each culture was transferred into TSB (Tryptic Soy Broth) media and cultured at constant shaking at 200 rpm/37°C until mid-log phase. Then bacterial suspension was adjusted by absorption measurement to a concentration of 10^8^/ml RPMI. 1 × 10^6^ sorted CD14^+^ monocytes were plated in 24-well plate format in 1 ml RPMI/10% FCS. Cells were infected with 1 × 10^6^ bacteria/ ml. After 2 h gentamicin (PAA Laboratories, Inc.) was added to a final concentration of 100 ng/ml. The next day cells were analyzed.

### Mouse model systemic inflammation

Twelve week old, male C57BL/6 mice were injected intraperitoneally with 1 mg/kg body weight LPS (Invivogen, San Diego, USA) or NaCl (control group). After 24 h, animals were euthanized and blood was taken and analyzed for DLL1 by ELISA assay (Abcam, Cambridge, UK).

### Flow cytometry

Monocytes were stimulated with 3 μg/ml recombinant soluble DLL1 (PeproTech, Hamburg, Germany) ±1 μM Oxaliplatin for 3 days. Additionally *E. coli* infected monocytes were treated with 2.5 μM DAPT on hour before infection. After killing bacteria by gentamicin, medium was changed and DAPT was added, again. Three days later monocytes were analyzed for surface expression of PD-L1 with antibody staining: α-PD-L1 (BD Biosciences, Heidelberg, Germany). Mean fluorescence was recorded using the FACS DIVA V 4.12 software on a FACS Canto (BD Biosciences). Overlays were performed with the Weasel v2.5 software (WEHI, Melbourne, VIC, Australia).

### Quantitative reverse transcription PCR (RT-qPCR)

Total RNA was extracted from 2 × 10^6^ cultured primary human monocytes using the high pure RNA isolation kit (Roche, Mannheim, Germany) according to the manufacturer's protocol. RNA preparations for miRNA expression analyses were carried out using the Ambion miRVana kit (Thermo Fischer Scientific, Karlsruhe, Germany) according to the manufacturer's instructions. In brief, 2 × 10^6^ cells were harvested by centrifugation, lysed and subjected to organic extraction with acid phenol:chloroform, followed by solid-phase extraction based on glass-fiber filters for efficient enrichment of small RNA species. RNA preparations enriched or not for small RNA species were then quantified by spectrophotometry (NanoDrop ND-100 Spectrometer, Peqlab, Erlangen, Germany) and equal amounts were reverse transcribed using the miScript II RT (Qiagen, Hilden, Germany) and Reverse Aid First Strand cDNA synthesis kits (Thermo Fischer Scientific, Karlsruhe, Germany), respectively. Obtained cDNA was used for quantitative PCR utilizing the “SYBR green ROX mix” (Thermo Fischer Scientific, Karlsruhe, Germany) and the following sequence-specific primers with regard to expression of protein coding genes: actin fwd 5′-AGA GCT ACG AGC TGC CTG AC-3′, actin rev 5′- AGC ACT GTG TTG GCG TAC AG-3′, JAG1 fwd 5′-CGGGATTTGGTTAATGGTTATC-3′, JAG1 rev 5′-ATAGTCACTGGCACGGTTGTAGCAC-3′, DLL1 fwd 5′-CCTACTGCACAGAGCCGATCT-3′, DLL1 rev 5′-ACAGCCTGGATAGCGGATACAC3′, HES1 fwd 5′-CTGAAGAAAGATAGCTCGCG-3′, HES1 rv 5′-ACTTCCCCAGCACACTT-3′, HEY1 fwd 5′-AGCCGAGATCCTGCAAGATGA-3′, HEY1 rv 5′-GCCGTATGCAGCATTTTCAG-3′.

### Enzyme-linked immunosorbent assay (ELISA)

Commercially available ELISA kits were utilized for detection of human IL-6, IL12, TNFα (BD OptEIA ELISA Set; BD Biosciences Pharmingen, Heidelberg, Germany), human DLL1 [DLL1 (Human) ELISA Kit, RayBiotech, Norcross Georgia, United States] and mouse DLL-1 (Abcam, Cambridge, UK). Assays were performed with cell-free supernatants according to the manufacturer's instructions. Absorbance was measured on a SUNRISE Absorbance reader (Tecan, Salzburg, Austria) and analyzed with Magellan software.

### Western blotting

2 × 10^6^ cells were harvested and washed with PBS. For whole cell lysates monocytes were lysed in 50 μl RIPA buffer (50 mM Tris-HCl, pH7.4; 1% Igepal; 0.25% sodium deoxycholate; 150 mM NaCl; 1 mM EDTA; 1 mM PMSF; 1 mg/ml each aprotinin, leupeptin, and pepstatin; 1 mM Na3VO4; and 1 mM NaF). Samples were vortexed and incubated 30 min on ice. Lysates were then cleared via centrifugation at 14,000 × g for 20 min. For generating nuclear extracts, cells were washed with cold PBS, resuspended in hypotonic buffer (20 mM Hepes pH 7.9, 10 mM KCl, 1 mM EDTA, 10% glycerol, 0.2% NP40, protease inhibitors) and stored on ice for 10 min. After centrifugation at 12,000 r.p.m. for 1 min at 4°C the supernatant containing the cytoplasmic fraction was removed. The pellet was resuspended in high-salt buffer (240 mM NaCl, 20 mM Hepes pH 7.9, 10 mM KCl, 1 mM EDTA, 20% Glycerol, 0.2% NP40, protease inhibitors), incubated for 30 min at 4°C and centrifuged for 10 min at 12,000 r.p.m. Equal amounts of whole cell or nuclear lysates were used for separation by SDS-PAGE (12.5%). After semi-dry transfer onto nitrocellulose membranes (Whatman Protran nitrocellulose membrane; neoLab, Heidelberg, Germany), the latter were blocked with 5% (w/v) BSA in TBS/0.1% (v/v) Tween-20 for 2 h at RT. Probing was performed with antibodies: Anti-p(y701) STAT3, anti-DLL1, anti-GAPDH (Cell Signaling Technology, Danvers, MA, USA) and anti B23 (Santa Cruz Biotechnology, Texas, USA). Detection was based on enhanced chemiluminescence (ECL; Perkin Elmer, Groningen, Netherlands).

### Quantification

Quantification of immunoblots was performed using the Image Analysis System Bioprofil (Fröbel, Germany) Bio ID software version 12.06. The intensity of signals were calculated against loading control and presented as percent of untreated samples.

### Statistical analysis

The comparison of two data groups were analyzed by Mann-Whitney U test with ^*^p ≤ 0.05.

### Ethics statement

This study (taking of blood samples from healthy donors and treatment of blood leukocytes with microbial stimuli) was carried out in accordance with the recommendations of the ethics committee of the Medizinische Fakultät Heidelberg with written informed consent from all subjects. All subjects gave written informed consent in accordance with the Declaration of Helsinki. The animal experiments were approved by the governmental animal ethics committee (Regierungspraesidium Karlsruhe, file number: 35-9185.81/G-132/15) and conducted according to international FELASA recommendations. The study was reviewed and approved by the ethics committee of Medizinische Fakultät Heidelberg.

## Author contributions

KH and DH designed the study with essential contribution from FU and MAW. DS, DH and UK performed the experiments. KH, DH, and FU prepared the manuscript. All authors discussed the results and implications and approved the manuscript.

### Conflict of interest statement

The authors declare that the research was conducted in the absence of any commercial or financial relationships that could be construed as a potential conflict of interest. The reviewer JM and handling Editor declared their shared affiliation.
