# Supplementary material for: The Interplay of Notch Signaling and STAT3 in TLR-Activated Human Primary Monocytes
Source: Front Cell Infect Microbiol. 2018 Jul 10;8:241. doi: 10.3389/fcimb.2018.00241 (PMC6048282; doi:10.3389/fcimb.2018.00241)
Supplement: Supplementary file 1 [file Data_Sheet_1.pdf]

Supplementary Material

The interplay of Notch signaling and STAT3 in TLR-activated human primary monocytes

Dagmar Hildebrand[1*], Florian Uhle[2], Delal Sahin[1], Ute Krauser[2], Markus Alexander Weigand[2], Klaus Heeg[1]

[1]Medical Microbiology and Hygiene, Centre for Infectious Diseases, Heidelberg University Hospital, Heidelberg, Germany

[2] Department of Anesthesiology, Heidelberg University Hospital, Heidelberg, Germany

***Correspondence:**
Dagmar Hildebrand: dagmar.hildebrand@med.uni-heidelberg.de

Supplementary Figures

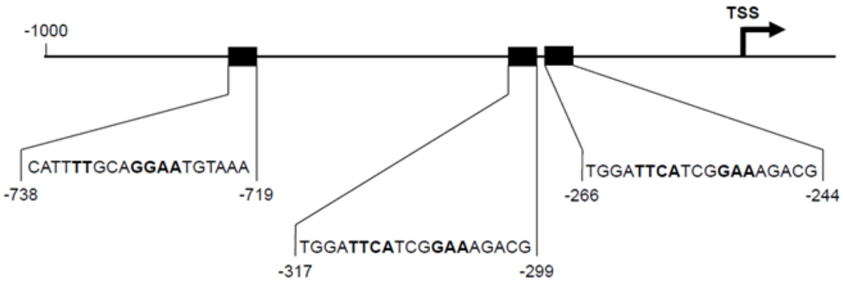

Supplementary Figure 1. STAT3 binding sites in the promoter region of DLL1.

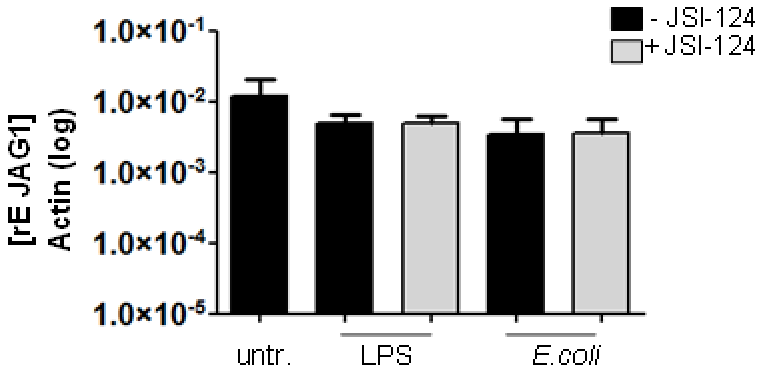

Supplementary Figure 2. STAT3-independent induction of LPS-stimulated JAG1. Human blood-derived monocytes were pretreated with STAT3 Inhibitor JSI-124 (200nM) for two hours before stimulation with LPS (100ng/ml) or infection with *E. coli* (10^6 bacteria per 10^6 monocytes/ml). After two hours bacteria were killed by gentamicin. RNA was isolated and cDNA produced. Induction of gene expression was analyzed by qRT PCR using a sequence-specific primer for JAG-1 (gene encoding Jagged-1) and SYBR Green Master mix. Actin was detected as endogenous control for normalization.
